# Targeted AAVP-based therapy in a mouse model of human glioblastoma: a comparison of cytotoxic versus suicide gene delivery strategies

**DOI:** 10.1038/s41417-019-0101-2

**Published:** 2019-05-27

**Authors:** Fernanda I. Staquicini, Tracey L. Smith, Fenny H. F. Tang, Juri G. Gelovani, Ricardo J. Giordano, Steven K. Libutti, Richard L. Sidman, Webster K. Cavenee, Wadih Arap, Renata Pasqualini

**Affiliations:** 1 Rutgers Cancer Institute of New Jersey and Division of Cancer Biology, Department of Radiation Oncology, Rutgers New Jersey Medical School, Newark, NJ USA; 2 Department of Biochemistry, Institute of Chemistry, University of São Paulo, São Paulo, São Paulo Brazil; 3 Karmanos Cancer Institute, School of Medicine and Department of Biomedical Engineering, College of Engineering, Wayne State University, Detroit, MI USA; 4 Rutgers Cancer Institute of New Jersey and Department of Surgery, Rutgers Robert Wood Johnson Medical School, New Brunswick, NJ USA; 5 Department of Neurology, Harvard Medical School, Boston, MA USA; 6 Ludwig Institute for Cancer Research, University of California-San Diego, La Jolla, CA USA; 7 Rutgers Cancer Institute of New Jersey and Division of Hematology/Oncology, Department of Medicine, Rutgers New Jersey Medical School, Newark, NJ USA

**Keywords:** Molecular biology, Gene delivery, Cancer

## Abstract

Glioblastoma persists as a uniformly deadly diagnosis for patients and effective therapeutic options are gravely needed. Recently, targeted gene therapy approaches are reemerging as attractive experimental clinical agents. Our ligand-directed hybrid virus of adeno-associated virus and phage (AAVP) is a targeted gene delivery vector that has been used in several formulations displaying targeting ligand peptides to deliver clinically applicable transgenes. Here we compared different constructs side-by-side in a tumor model, an orthotopic model of xenograft human glioblastoma cells stereotactically implanted in immunodeficient mice. We have used divergent therapeutic strategies for two AAVP constructs, both displaying a double-cyclic RGD4C motif ligand specific for alpha V integrins expressed in tumor vascular endothelium, but carrying different genes of interest for the treatment of intracranial xenografted tumors. One construct delivered tumor necrosis factor (*TNF*), a purely cytotoxic gene for antitumor activity (RGD4C-AAVP-*TNF*); in the other construct, we delivered *Herpes simplex* virus thymidine kinase (*HSVtk*) for in tandem molecular-genetic imaging and targeted therapy (RGD4C-AAVP-*HSVtk*) utilizing ganciclovir (GCV) for a suicide gene therapy. Both AAVP constructs demonstrated antitumor activity, with damage to the tumor-associated neovasculature and induction of cell death evident after treatment. In addition, the ability to monitor transgene expression with a radiolabeled HSVtk substrate pre and post GCV treatment demonstrated the theranostic potential of RGD4C-AAVP-*HSVtk*. We conclude that targeted AAVP constructs delivering either cytotoxic *TNF* or theranostic *HSVtk* followed by suicide gene therapy with GCV have comparable preclinical efficacy, at least in this standard experimental model. The results presented here provide a blueprint for future studies of targeted gene delivery against human glioblastomas and other brain tumors.

## Introduction

Glioblastomas are lethal intracranial tumors of the central nervous system characterized by high morbidity and mortality due to their high tumor cell proliferation rate and marked neovascularization, allowing them to infiltrate crucial structures in the brain [1]. The delicate nature of the brain, combined with structural constraints such as the blood–brain barrier and the presence of tightly regulated, isolated physiological processes, including the glymphatic system [2], limits clinical options, particularly drug delivery, for brain tumors [3]. The current standard-of-care for human glioblastoma consists of radiotherapy, often in concert with chemotherapy, which offers only modest benefits and remains essentially palliative, and, although maximum surgical resection may be recommended, once the tumor cells infiltrate the surrounding brain parenchyma, surgery is essentially ineffective without producing substantial neurological injury [1]. The options for recurrent tumors are even more limited. Therefore, there is a grave unmet need for innovative treatment options. Given that targeted gene therapy is undergoing a renascence due to the potential efficacy of oncolytic viruses and other viral vectors in malignant gliomas [4] and other tumor types [5], we hypothesized that a new class of ligand-directed gene therapy vector might be a promising strategy for overcoming the barriers to treat human glioblastoma.

Over the past decade, our group has advanced a targeted hybrid vector containing *cis*-genomic elements from adeno-associated virus (AAV) and single-stranded M13-derived bacteriophage displaying tumor-homing ligand peptide motifs to target endothelial and tumor cell surface receptors and to mediate selective internalization of ligand-directed viral particles [6]. In the hybrid AAV/phage (termed AAVP) system, there is no AAV capsid formation and the ligand peptides displayed on the capsid of the phage particle allow homing to tumor-specific receptors. Such hybrid vectors can be engineered to deliver the therapeutic and/or theranostic transgenes directly to tumors after systemic intravenous (i.v.) administration. Indeed, we have previously shown the ligand-directed delivery of a tumor necrosis factor (*TNF*) transgene to tumors in the setting of human melanoma xenografts [7, 8], a transgenic model of pancreatic neuroendocrine tumors [9], and even in spontaneous native tumors in pet dogs [10] consistently produced antitumor effects without evidence of off-target side effects, a major concern of nontargeted systemic TNF therapy, which is known for severe toxicity [11]. AAVP has also been engineered to contain a *Herpes simplex* virus-1 thymidine kinase (*HSVtk*) transgene, which serves as both a reporter for clinically applicable positron emission tomography (PET) imaging with HSVtk-specific radiolabeled nucleoside analogs, such as [^18^F]-FEAU, and/or a suicide gene therapy strategy when combined with ganciclovir (GCV). This theranostic convergence of molecular-genetic imaging and targeted therapy has shown promise in preclinical models of several solid tumor types [6, 12–15].

The broad utility of targeted AAVP constructs displaying the RGD4C motif (a double-cyclic sequence CDCRGDCFC) targeting the αv integrin subunit has previously been established with AAVP constructs containing either *TNF* (RGD4C-AAVP-*TNF*) [7, 8, 10] or *HSVtk* (RGD4C-AAVP-*HSVtk*) [6, 14] transgenes. However, we have yet to ascertain the relative efficacy of each transgene in the same tumor model. Since integrin subunit αv is highly expressed both in tumor cells and in angiogenic vasculature in glioblastomas [16–18], here we evaluate two parallel strategies for ligand-directed therapy with a cytotoxic agent (TNF) versus a theranostic (HSVtk) gene delivery followed by suicide gene therapy with GCV in the same orthotopic mouse model of human glioblastoma with RGD4C-directed AAVP vectors.

Our results demonstrate that RGD4C-AAVP-*TNF* therapy reduces tumor size in a dose-dependent manner, disrupts tumor blood vessels, and works through an apoptotic pathway. Similarly, with RGD4C-AAVP-*HSVtk* administration followed by GCV dosing, experimental tumors showed blood vessel damage and marked evidence of apoptosis. We conclude that the magnitude of tumor response was comparable and roughly equivalent overall survival of the experimental cohorts between the two prototypes evaluated. However, by administering a radiolabeled HSVtk substrate, these tumors could also be imaged horizontally during the course of the study to evaluate transgene expression over time, a transgene-specific tool potentially useful for timing GCV and for evaluating tumor response, a feature not currently available with the cytotoxic TNF vector.

## Materials and methods

### Animals

We used 8-week-old female nude (*nu/nu*) mice. All animal procedures were reviewed and approved by the Institutional Animal Care and Use Committee of the appropriate institution.

### Preparation of targeted viral particles

Design and construction of targeted AAVP vectors has been reported [6, 7]. Targeted AAVP particles were produced and verified with standard experimental protocols [19, 20]. Briefly, following infection of k91Kan *Escherichia coli*, cultures were grown overnight, precipitated with NaCl/PEG, and resuspended in phosphate-buffered saline. Immediately before its use, AAVP was carefully re-titrated by k91Kan *E. coli* infection. Serial dilutions were plated on LB agar plates containing tetracycline and kanamycin as selectable markers. Transducing units (TU) were determined by colony counting [19, 20].

### Cell culture

U87-MG human glioblastoma cells were originally obtained from American Type Culture Collection (ATCC; Manassas, VA) and cultured as described [15]. ATCC uses various approaches to verify cell line identity of cell lines and ensure no contaminants are present. Cells were free of mycoplasma upon arrival and were tested periodically thereafter.

### Orthotopic human glioblastoma intracranial xenografts

A guide-screw system was used to implant human glioma cells into the mouse brain, as described [15, 21]. Animals were kept warm after implantation to recover from the anesthetic and then allowed to move freely. No randomization methods were applied, no blinding was done for any experiments, and all animals were included in the analyses.

### Therapeutic targeted AAVP administration in glioma-bearing mice

Orthotopic brain tumor-bearing animals (*n* = 5 mice/experimental group, which was chosen to allow for a detectable effect size) received doses of RGD4C-AAVP-*TNF* (three concentrations of vector were tested: 5 × 10^9^ TU, 5 × 10^10^ TU or 5 × 10^11^ TU i.v. per mouse) or negative control on days 5, 8, 11, and 14 after tumor implantation. Whole brains, including any intracranial tumors, were collected 4 days after the final dose was administered for analysis.

### Targeted suicide gene therapy and molecular-genetic imaging

Seven days after tumor implantation, orthotopic brain tumor-bearing animals (*n* = 5 mice/experimental group) received a single dose (5 × 10^11^ TU i.v. per mouse) of RGD4C-AAVP-*HSVtk*. Either GCV (80 mg/kg/d i.p.) or saline control was administered daily from days 18 to 23 after tumor implantation. One day after the last treatment with GCV, animals were killed and whole brains removed for histopathological analysis. To evaluate *HSV*tk gene expression, PET imaging was performed 2 h after i.v. administration of the radiolabeled nucleoside analog [^18^F]-FEAU [22]. PET imaging was performed with an Inveon micro-PET/CT scanner (Siemens Preclinical Solution). Glioma-bearing mice were anesthetized (with isoflurane 2% in 98% oxygen), and their temperature was kept at 38 °C with a heat lamp. Fully 3D list mode data were collected by using an energy window of 350–750 keV and a time window of 6 ns. Images were reconstructed by a 2D ordered subsets expectation maximization algorithm. PET image analyses were performed with vendor software ASIPro 5.2.4.0 (Siemens Preclinical Solutions).

### Immunohistochemistry

CD31 staining was performed on an automated immunohistochemical autostainer (Lab Vision Corp.) using a rabbit polyclonal anti-CD31 (AbCam, catalog # ab28364) and secondary HRP-conjugated anti-rabbit IgG (ThermoFisher Scientific, catalog # G-21234).

### Immunofluorescence

Detection of apoptosis in paraffin-embedded specimens was performed with the FragEL DNA Fragmentation Detection Kit (MilliporeSigma, catalog # QIA391EA).

### Expression of integrin αv subunit in human glioblastoma

Protein expression data generated within the Human Protein Atlas [23] (data available from v18.proteinatlas.org).

### Statistics

Graphpad Prism software v.5.03 and Microsoft Excel were used to graph data as mean ± SD and to calculate *P* values by using homoscedastic (one-tailed) Student’s *t* tests. *P* < 0.05 were considered statistically significant.

## Results

### RGD4C-AAVP-TNF targets experimental glioblastoma in vivo and has dose-dependent effect

**Fig. 1:**
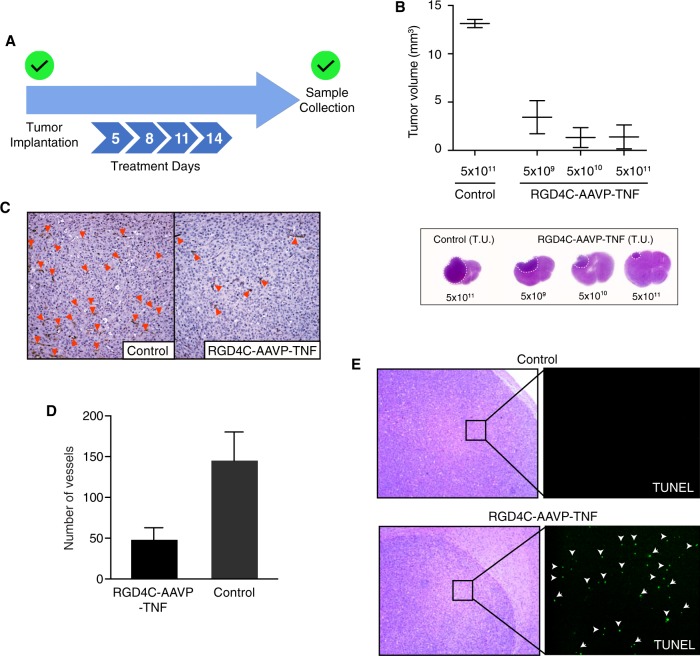
Tumor growth inhibition after RGD4C-AAVP-*TNF* administration. **a** Experimental design (*n* = 5 mice/experimental group). Doses of 5 × 10^9^, 5 × 10^10^, and 5 × 10^11^ TU of RGD4C-AAVP-*TNF* were tested; samples were collected after 4 i.v. administrations. **b** Tumor volume reported as mean ± standard deviation. Representative images of hematoxylin and eosin (H&E) staining demonstrating a dose-dependent decrease in tumor size after targeted AAVP treatment. **c** CD31 staining on tumor sections of animals given targeted AAVP or negative control. A representative image from the 10^9^ treatment cohort is shown. Arrows point to disrupted blood vessels. **d** Blood vessel quantification in treated group and control reported as mean ± standard deviation (*n* = 10 fields/tumor). **e** TUNEL staining of tumors from treated and control animals. Arrowheads point to apoptotic cells

We first set out to evaluate the translational potential of RGD4C-AAVP-*TNF* in an experimental orthotopic preclinical model of human glioblastoma cells stereotactically implanted in immunodeficient mice. In a repeat dose study, we tested three doses of RGD4C-AAVP-*TNF* (5 × 10^9^, 5 × 10^10^, and 5 × 10^11^ TU), administered on days 5, 8, 11, and 14 after tumor intracranial implantation (Fig. 1a). Compared to animals in the negative control group we noted extensive tumor regression in mice treated with systemic doses of RGD4C-AAVP-*TNF* with an evident dose-dependent effect. Animals in the negative control group showed no tumor regression (Fig. 1b). Staining with an anti-CD31 antibody revealed disrupted tumor blood vessels within the intracranial tumors of animals in the RGD4C-AAVP-*TNF* treated groups, a finding not observed in the tumors from negative control animals (Fig. 1c, d). Furthermore, terminal deoxynucleotidyl-transferase-mediated dUTP-biotin nick end labeling (TUNEL) staining detected cells undergoing apoptosis in the tumors from animals treated with RGD4C-AAVP-*TNF*, while no apoptosis was observed in tumors from mice in the negative control group (Fig. 1e). Interestingly, the U87 cell line has been shown to express integrin subunit αv for targeting with RGD4C peptide ligands [24, 25], so while endothelial cells are likely the major source of TUNEL staining, positive staining likely represented tumor cell death as well. Combined, these results demonstrate that RGD4C-AAVP-*TNF* treatment of orthotopic glioma-bearing mice markedly suppressed tumor growth in a dose-dependent manner after sufficient TNF production to disrupt the tumor vasculature and to induce apoptosis in tumor cells.

### Theranostic tumor targeting and transgene delivery in an orthotopic mouse model of glioma

**Fig. 2:**
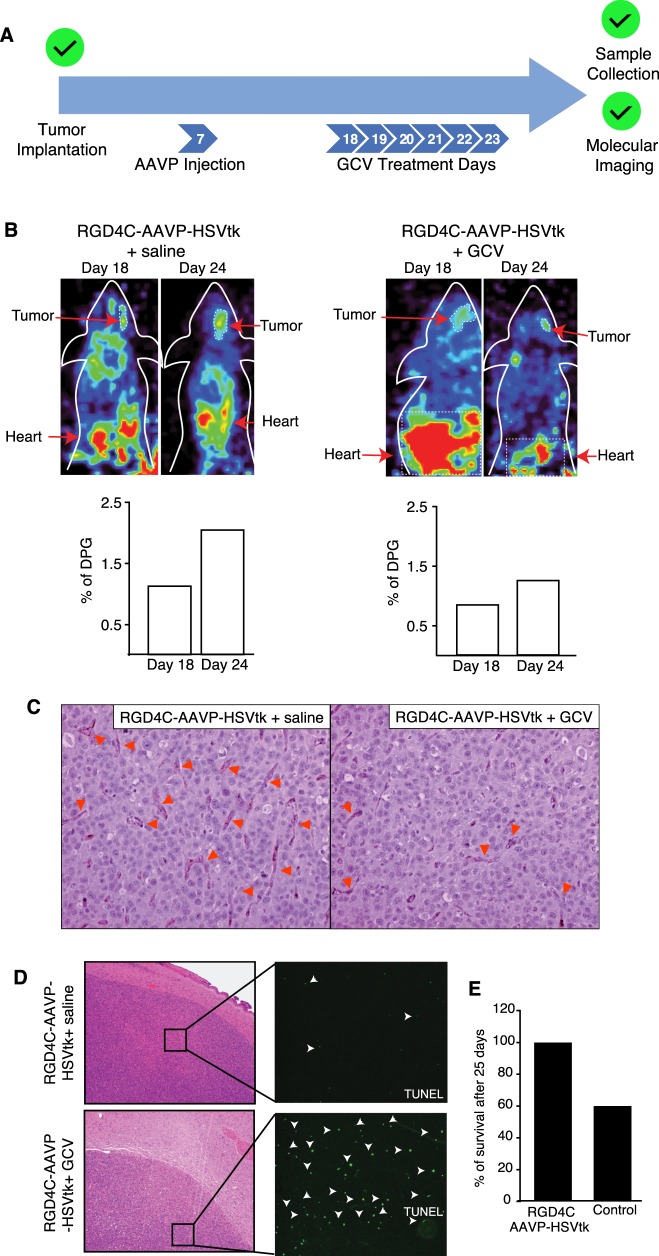
Targeted therapy and molecular-genetic imaging of human malignant glioma xenografts with RGD4C-AAVP-*HSVtk*. **a** Experimental design (*n* = 5 mice/experimental group). After tumor implantation, 5 × 10^11^ TU AAVP was administered i.v., a first round of imaging was completed to confirm transgene expression followed by daily doses of GCV or saline, and a second imaging study was completed to evaluate tumor response. **b** PET imaging in tumor-bearing mice after systemic delivery of RGD4C-AAVP-*HSVtk*. PET images show [^18^F]-FEAU accumulation before (Day 18) and after (Day 24) treatment with GCV or saline control. **c** Histopathological analysis of tumors. CD31 staining reveals the presence of disrupted blood vessels in the targeted AAVP plus GCV treatment group (indicated by arrows). **d** TUNEL staining indicating the presence of apoptotic cells (arrowheads) following AAVP administration and treatment with either GCV or saline control. **e** Percent of survival after 25 days of treatment

Molecular-genetic imaging is currently not possible with targeted AAVP-*TNF* vectors, so routine clinical MRI or PET/CT would have to be used in parallel with cytotoxic therapy to evaluate tumor response over time without a corresponding measurement of transgene expression. This is not the case with AAVP constructs designed to deliver a theranostic *HSVtk* transgene, which allow the merging of molecular-genetic imaging and targeted ablation of glioblastoma cells and tumor vascular endothelial cells through a suicide gene therapy approach. Toward that goal, we administered a single dose of RGD4C-AAVP-*HSVtk* to cohorts of the same orthotopic mouse model of human glioblastoma 7 days after cell implantation. To confirm tumor transduction and ascertain the magnitude of HSVtk expression, PET scan-based imaging with [^18^F]-FEAU was performed 10 days after AAVP administration. After this baseline PET image, animals in the treatment group were given GCV daily for 6 days, while the negative control group received a daily dose of saline solution. One day after the final GCV dose, animals that survived 24 days post tumor implantation underwent a post-treatment PET scan, were killed, and brains including any tumors were recovered for pathological examination (Fig. 2a). After 6 days of systemic GCV or saline treatment, PET scan imaging of [^18^F]-FEAU showed a marked decrease in the levels of HSVtk expression in brain tumors from animals in the GCV treatment group, while images from animals in the negative control group treated with saline solution showed increased probe expression (Fig. 2b). As with the AAVP-*TNF* study, response to treatment was assessed by CD31 blood vessel staining and TUNEL to detect apoptotic cells. CD31 staining revealed the presence of disrupted blood vessels in animals in the GCV treatment group, and this pattern was not observed in negative control mice treated with RGD4C-AAVP-*HSVtk* and saline (Fig. 2c). Furthermore, in tumors from mice treated with RGD4C-AAVP-*HSVtk* followed by GCV, TUNEL staining detected apoptosis preferentially observed within the tumor center relative to the outer tumor rim, while no apoptosis above background level was observed in tumors from negative control mice (Fig. 2d). Interestingly, we also noted that no deaths were recorded in the RGD4C-AAVP-*HSVtk* plus GCV treatment group, while only about 60% of animals in the negative control group were alive after the prolonged 25-day experimental timeline, which, due to the two pre- and post-treatment imaging steps in our experimental design, was extended toward the upper limits of this aggressive intracranial tumor model (Fig. 2e) [21]. Together, these results establish that RGD4C-AAVP-*HSVtk* can localize to brain tumors for transduction in the setting of a preclinical model of glioblastoma, and HSVtk expression can be serially monitored over time to enable a theranostic (“see and treat, treat and see”) translational approach for diagnostics and to determine tumor response to GCV treatment.

### Integrin subunit αv is highly expressed in human glioblastoma

**Fig. 3:**
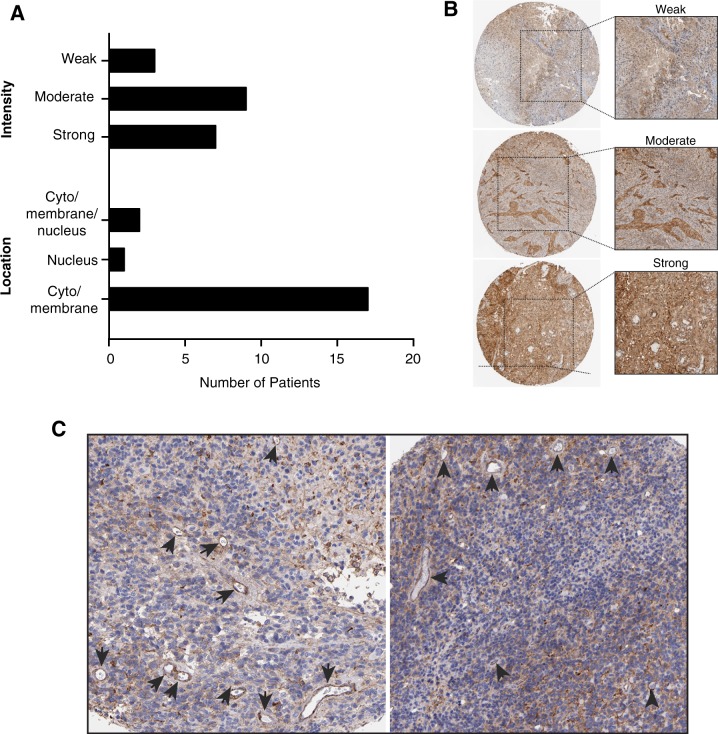
Expression of integrin subunit αv in human glioblastoma visualized by immunohistochemistry (image credit: Human Protein Atlas). **a** Intensities of integrin subunit αv staining were deemed strong (3+), moderate (2+) or weak (1+). Presence of integrin αv in tumor tissue was predominant in the cytoplasm and membrane of cells, with minimum staining detected in the nucleus, as described in the text. **b** Representative images of staining intensities in tumor samples. **c** Integrin αv expression was also found in the tumor-associated neovasculature. Arrows point to blood vessels

Finally, we used a publicly available database (The Human Protein Atlas) to assess expression of the target integrin subunit αv in human gliomas and to support potential clinical applications of AAVP constructs. A cohort of human malignant gliomas (*n* = 25) with varying tumor grades was quantitatively analyzed. Intensity of staining was denoted as strong (3+), moderate (2+) and weak (1+) (Fig. 3a, b). In ~28% of the cases studied (7 patients, aged from 10 to 72 years old) showed strong integrin subunit αv expression, whereas most patients had moderate (*n* = 13, 52%) and low (*n* = 5, 20%) staining intensities (Fig. 3a). Location of integrin αv expression was mostly restricted to cytosol and membrane (80%), with five cases (20%) of positive nuclear expression (Fig. 3a). Most strikingly, marked expression of the integrin αv subunit was clearly detected in blood vessels associated with tumors (Fig. 3c, arrows), thus supporting and affirming our programmatic efforts toward clinical translation of one or both of our targeted gene vector constructs.

## Discussion

To ascertain the translational potential of the RGD4C ligand motif, we analyzed the efficacy of two AAVP constructs in an orthotopic model of glioblastoma. Gratifyingly, both the cytotoxic and theranostic versions of targeted AAVP showed antitumor effects with damage to the tumor-associated vasculature and evidence of apoptotic cells. However, one must consider pros and cons of targeted delivery of *TNF* or *HSVtk* plus GCV as we design further translational studies. The cytokine TNF, for example, is an effective antitumor cytotoxic and may be used as a single-agent in repeat dose studies, greatly lessening the complexity of a treatment scheme. Imaging studies are routinely done to evaluate tumor response serially over time in glioblastoma patients [1], so tumor size and metabolism could be measured in concert with an AAVP delivering *TNF*, but not gene expression itself. Alternatively, *HSVtk* provides an opportunity to evaluate molecular transgene expression serially with a radiolabeled probe. The combination of targeted delivery of an *HSVtk* transgene, molecular-genetic imaging with a radiolabeled probe to noninvasively evaluate gene expression, and GCV suicide gene therapy might ultimately lead to a complex, yet elegant clinical trial design that could be tailored to individual patients who would be monitored over time to non-invasively monitor *HSVtk* gene expression. Once maximum expression is reached, GCV dosing could begin and both the transgene and tumor monitored over time. While persuasive, this may be a harder trial design to evaluate in patients, with multiple moving parts to consider in a clinical setting. Because gliomas are routinely imaged through MRI, targeted AAVP-*TNF* vectors would seem to have a straightforward clinical path. This is one reason why RGD4C-AAVP-*TNF* was chosen for translational application (phase zero to phase II) in a large cohort of pet dogs with native tumors [10] and it is currently ongoing Good Manufacturing Practice (GMP) production for a first-in-human trial (unpublished results).

There are numerous gene therapy approaches being pursued in gliomas, recently reviewed by Caffery et al. [26]. Clinical trials are underway for viral and nonviral vectors and encompassing suicide gene therapy, immunotherapy, and delivery of oncolytic or tumor suppressor genes. Retroviral and adenoviral vectors, mainly utilizing HSVtk or cytoside deaminase suicide gene therapy, are the most common therapeutic approaches evaluated in patients, often with only modest benefits in trials against the current standard of care. Non-viral approaches, such as liposomal formulations, attempt to overcome the lack of targeting capabilities and the immunogenicity of the viral vectors [26]. One formulation of functionalized liposomes encapsulating p53 plasmid DNA [27] is being evaluated in a phase II study (NCT02340156); the wild-type p53 serves to make the tumors susceptible to chemotherapy, a noteworthy advance for this notoriously chemoresistant tumor type, but still requiring patients to be exposed to chemotherapeutic drugs and their associated toxicities.

Many studies succumb to a few commonly identified roadblocks to gene therapy against glioblastomas. Notably, poor transduction efficiency is a frequent occurrence, and several reasons have been postulated for inefficient cell transduction, including poor penetrance after intratumoral injections and lack of a viral receptor for viral vector gene therapy approaches [28]. We attempt to avoid these particular limitations by using systemic administration and a ligand-directed viral particle targeted to the αv integrins. Malignant gliomas often demonstrate a marked upregulation of vascular endothelial growth factor and other angiogenic markers, including vascular integrins, rendering them potentially susceptible to antiangiogenic therapies [1, 29]. As is typical of many targeting paradigms, including phage-based approaches, protein upregulation is likely insufficient for targeting, and accessibility to circulating ligands by way of receptor localization to the membrane is required for internalization and, in the case of targeted AAVP, transduction of the cell [30]. The RGD4C peptide and its corresponding binding partners, αv integrins expressed in tumor-associated endothelial cells of the neovasculature, are well characterized and common therapeutic targets in gliomas [24, 25, 31]. For example, trials with a cyclic RGD peptide motif that functions as a small molecule inhibitor of αv integrins showed both safety and some efficacy after systemic administration, although the results overall have been relatively modest [3, 32]. Importantly, these efforts demonstrate that integrins are a viable target for gene delivery approaches. If receptor expression might be a valid concern, HSVtk-based molecular-genetic imaging would confirm receptor expression and cell transduction.

Early stage clinical trials utilizing either HSVtk or TNF in various viral constructs have been reported, which is encouraging for our ongoing translational efforts. Many of these trials have demonstrated both safety and some efficacy in patients with malignant glioma [4]. The combination of imaging and therapy with HSVtk substrates after nontargeted intratumoral administration was described in glioma patients in the early 2000s and, while seemingly safe, the authors hypothesized that a low tumor proliferation index hindered transduction and gene expression in a sufficient number of cells [33]. While this justification is likely true, we would argue that, in addition to a second round of gene administration after failure to visualize substantial gene expression, the inclusion of a targeting moiety could expand the number of transduced cells to a more effective number. Oncolytic viruses carrying the RGD4C peptide motif have also been studied in multiple phase I/II trials. Safety and efficacy, along with some dramatic responses have been reported [34]. More reports are expected soon, since there are clinical trials underway or recently completed [4]. Unfortunately, the vectors used in these trials require either intratumoral injection or delivery to the tumor periphery post resection, which greatly increases the level of technical complexity.

Furthermore, although reproducibly sufficient gene expression has been found in several studies utilizing the AAVP gene delivery vector, transduction efficiency is still notably poor. One could speculate that this is not a fatal flaw in our case: both TNF and the combination of HSVtk plus GCV work via a bystander effect, maximizing the effect of even low transduction efficiency. In the case of TNF, we must be careful to avoid tipping the delicate balance of TNF signaling from apoptotic to proliferative due to intrinsic factors in the tumor microenvironment [35]. In this respect, inefficient transduction may not be a disadvantageous attribute of the targeted AAVP system; inclusion of a moiety that tips the balance toward apoptosis may actually be beneficial as it might lead to a synergistic cytotoxic effect [8]. Finally, while a difference in outcomes is rather challenging to precisely and accurately document in the setting of this very aggressive preclinical model with a rapid and uniformly lethal outcome, we observed a roughly comparable overall survival in side by side experiments (data not shown). Those experimental limitations and caveats notwithstanding, development of targeted AAVP vectors for both cytotoxic (TNF) versus suicide gene therapy (HSVtk/GCV) delivery will continue to be pursued toward a first-in-human trial. If successful, this first direct comparison of antitumor activity in a single intracranial tumor model will be considered a relevant milestone for the ongoing study design and translational applications of targeted AAVP-based imaging and therapy.
